# Rapamycin Improves Palmitate-Induced ER Stress/NF***κ***B Pathways Associated with Stimulating Autophagy in Adipocytes

**DOI:** 10.1155/2015/272313

**Published:** 2015-01-14

**Authors:** Jiajing Yin, Liping Gu, Yufan Wang, Nengguang Fan, Yuhang Ma, Yongde Peng

**Affiliations:** Department of Endocrinology, Shanghai First People's Hospital, Shanghai Jiao Tong University, 100 Haining Road, Shanghai 200080, China

## Abstract

Obesity-induced endoplasmic reticulum (ER) stress and inflammation lead to adipocytes dysfunction. Autophagy helps to adapt to cellular stress and involves in regulating innate inflammatory response. In present study, we examined the activity of rapamycin, a mTOR kinase inhibitor, against endoplasmic reticulum stress and inflammation in adipocytes. An *in vitro* model was used in which 3T3-L1 adipocytes were preloaded with palmitate (PA) to generate artificial hypertrophy mature adipocytes. Elevated autophagy flux and increased number of autophagosomes were observed in response to PA and rapamycin treatment. Rapamycin attenuated PA-induced PERK and IRE1-associated UPR pathways, evidenced by decreased protein levels of eIF2*α* phosphorylation, ATF4, CHOP, and JNK phosphorylation. Inhibiting autophagy with chloroquine (CQ) exacerbated these ER stress markers, indicating the role of autophagy in ameliorating ER stress. In addition, cotreatment of CQ abolished the anti-ER stress effects of rapamycin, which confirms the effect of rapamycin on ERs is autophagy-dependent. Furthermore, rapamycin decreased PA-induced nuclear translocation of NF*κ*B P65 subunit, thereby NF*κ*B-dependent inflammatory cytokines MCP-1 and IL-6 expression and secretion. In conclusion, rapamycin attenuated PA-induced ER stress/NF*κ*B pathways to counterbalance adipocytes stress and inflammation. The beneficial of rapamycin in this context partly depends on autophagy. Stimulating autophagy may become a way to attenuate adipocytes dysfunction.

## 1. Introduction

Over recent decades, it is clear that obesity is associated with the activation of endoplasmic reticulum (ER) stress signalling and inflammatory pathways, which contributes to obesity-related metabolic syndrome and type 2 diabetes [1]. At the level of adipose tissue, systemic inflammation may be initiated by adipocytes dysfunction [2]. Hypertrophy of adipocytes is considered a key event associated with a loss of insulin sensitivity in both lean and obese conditions [3]. Individuals with larger adipocytes typically have elevated proinflammatory factors including leptin, IL-6, IL-8, monocyte chemoattractant protein-1 (MCP-1), and reduced levels of the insulin-sensitivity-related adiponectin and IL-10 [4]. Saturated fatty acids (SFA) are systemically elevated in diet-induced obesity and trigger ER stress, which has been proposed as the immediate cause of chronic inflammation and reduction of insulin action [5]. ER stress signalling referred to as the unfolded protein response (UPR) is triggered by three downstream proteins: PKR-like eukaryotic initiation factor 2 kinase (PERK), activating transcription factor 6 (ATF6), and inositol requiring 1*α* (IRE1*α*). The PERK-elongation initiation factor 2*α*-activating transcription factor 4 (ATF4) signaling pathway connects ER stress to C/ebp homologous protein (CHOP) and contributes to ER stress-induced apoptosis [6]. IRE1*α* mediated recruitment of tumor necrosis factor receptor-associated factor 2 and signal-regulated kinase 1/C-jun N-terminal kinase (JNK). ER stress intersects with many different inflammatory signalling pathways, such as NF*κ*B signalling and contributes to the increase of inflammatory cytokines [7].

Autophagy or “self-eating” is an evolutionarily conserved lysosome-dependent system in eukaryotes that regulates the turnover of cellular proteins and organelles. During autophagy, target proteins or organelles are delivered into double-membrane autophagosomes for lysosomal degradation. Indeed, autophagy has been closely linked to control of innate and adaptive immune responses in part by regulating cytokine production [8, 9] and also linked to ER stress/UPR pathway [10]. Deficient autophagy by suppression of autophagy related gene 7 (ATG7) renders hepatocytes vulnerable to ER stress and insulin resistance, and conversely restoration of hepatic autophagy be means of ATG7 overexpression can improve insulin sensitivity [11].

The nutrient-sensing kinase mammalian target of rapamycin complex 1 (mTORC1) is an important regulator of autophagy [12]. Treatment with rapamycin further stimulated autophagy by increased autophagosome formation and enhancement of autophagosome-lysosome fusion [13]. The mTORC1 is composed of mTOR, regulatory associated protein of mTOR (Raptor) and mLST8, and is sensitive to rapamycin. mTORC1 is one of the key regulators for cell metabolism through mTORC1-mediated direct phosphorylation of ribosomal p70S6 kinase (p70S6K) and eukaryotic initiation factor 4E- (eIF4E-) binding protein. Rapamycin has been suggested for the treatment of mTORC1-related diseases, including cancer, cardiovascular diseases, and metabolic disorders [14]. Interestingly, these diseases are also considered to the ER stress-related disorders. In Akita *β* cells, rapamycin reduces ER stress via autophagy and prevents diabetes progression in Akita mice [13].

To the best of our knowledge, little is known about how ER stress and autophagy interacts as well as with inflammation in adipocytes. In this study, we set out to examine the role of rapamycin in palmitate-induced adipocytes ER stress and inflammation. Study shows that the benefit effect of rapamycin may be due to activation of autophagy and the inhibition of ER stress/NF*κ*B signalling pathway.

## 2. Materials and Methods

### 2.1. Reagents

Fetal bovine serum (FBS), culture media DMEM, TRIzol reagent were from Gibco Invitrogen (Grand Island, NY, USA), Superscript III First-strand Synthesis System was from Promega (Madison, USA). Tunicamycin (Tm), thapsigargin (TG), and rapamycin (RAP) were purchased from Merck Bioscience (Darmstadt, Germany). Palmitate, (PA P0500), BSA (albumin, endotoxin), CQ (chloroquine), MDC (monodansylcadaverine), 4-phenyl butyrate (4-PBA), and BaY11-7082 were from Sigma-Aldrich (St. Louis, MO, USA). Bradford reagent, acrylamide, and immunoblot PVDF membrane were from Roche (Roche Diagnostics, Barcelona, Spain). Immobilon Western Chemiluminescent HRP substrate was purchased from Millipore (Billerica, MA, USA). Anti-LC3, ATF4, CHOP, P-eIF2*α*, p-JNK, JNK, NKP65, *β*-actin, LAMIN A/C, phospho-S6, and GAPDH were from either CST (Cell Signaling Technology, Beverly, MA, USA) or Santa Cruz Biotechnology, Inc. (Santa Cruz, CA, USA).

### 2.2. Cell Culture and PA Treatment

3T3-L1 cells were obtained from the American Type Culture Collection (Rockville, MD). Cells were seeded and fed every 2 days in DMEM containing 25 mM glucose supplemented with 50 U/mL penicillin, 50 *μ*g/mL streptomycin, 100 mM MEM sodium pyruvate, and 10% FBS. Cells were grown under 5% CO_2_ at 37°C. At confluence, differentiation was induced by addition of medium containing 500 mM isobutylmethylxanthine (Sigma), 250 mM dexamethasone (Sigma), and 1.7 mM insulin. After 48 h, this mixture was replaced with fresh medium, and this was changed every 2 days. On days 6–8 after the induction of adipocyte differentiation, cells were used for experiments. PA/BSA conjugates were prepared as described previously [15].

### 2.3. Electron Microscopy

After the indicated treatments, mature 3T3-L1 adipocytes were fixed in phosphate buffer (pH 7.4) containing 2.5% glutaraldehyde and 2% paraformaldehyde at room temperature for 60 min. Cells were postfixed in 1% OsO_4_ at room temperature for 60 min, dehydrated through graded ethanol solutions, and embedded in Quetol 812 (Nissin EM Co., Tokyo, Japan). Areas containing cells were block-mounted and cut into 70 nm sections that were stained with uranyl acetate (saturated aqueous solution) and lead citrate and examined with a transmission electron microscope (H-7100; Hitachi, Ibaraki, Japan).

### 2.4. Immunofluorescence

We recently developed an assay that allows quantitation of autophagy by measuring incorporation of the autofluorescent marker monodansylcadaverine (MDC) [16]. Briefly, cells were incubated in full or starvation nutrient medium and labeled with 0.05 mM MDC in PBS at 37°C for 10 min. After incubation, cells were washed four times with PBS and resuspended in 10 mM Tris-HCl pH 8 containing 0.1% Triton X-100. Intracellular MDC was measured by fluorescence photometry (excitation, 380 nm; emission, 525 nm) in a Packard Fluorocount microplate reader.

### 2.5. Western Blotting

Cells were washed with PBS and lysed in lysis buffer (0.5% Triton X-100, 10 mM HEPES pH 7.9, 50 mM NaCl, 100 mM EDTA, and 0.5 M sucrose) containing 0.1% protease inhibitor cocktail (Roche Diagnostics, Barcelona, Spain). Lysates were then incubated on ice for 30 min and centrifuged at 8,000 ×g for 10 min. Equal amounts of protein were subjected to SDS-PAGE (10–15%), transferred to polyvinylidene difluoride membranes, and immunoblotted with primary antibody. Membranes were washed with PBS-Tween-20 and incubated with peroxidase-conjugated secondary antibody. Protein bands were detected using an ECL Plus kit (Amersham Biosciences Corp., Piscataway, NJ, USA).

### 2.6. Quantitative Real-Time PCR Analysis

Total RNA was extracted using TRIzol reagent and reverse-transcribed using a SuperScript III First-Strand Synthesis System for qPCR following manufacturer's instructions. qPCR was performed with an ABI 7900 sequencer (Life Technologies, Carlsbad, CA, USA) using the SYBR Green method and d(N)_6_ random hexamer with primers from Invitrogen. Thermocycling parameters were 95°C for 10 min, 40 cycles of 95°C for 15 s, 60°C for 1 min. Each sample was run in triplicate and normalized against* 36B4* RNA. Fold changes were determined using the delta delta Ct method. Primers used are listed in Table 1.

### 2.7. Enzyme-Linked Immunosorbent Assay

Cytokines IL-6 in cell culture supernatants was measured by enzyme-linked immunosorbent assay, according to the manufacturer's protocols (ABCAM, CA, USA).

### 2.8. Statistical Analysis

Results are expressed as means ± SEM. Comparisons of a single variable in >2 groups were analyzed by one-way ANOVA followed by Bonferroni's multiple comparison tests. Statistical analysis was performed using the paired and unpaired *t*-test between two groups, using SPSS 12.0 (SPSS Inc., Chicago, IL, USA). Values of *P* < 0.05 were considered statistically significant.

## 3. Results

### 3.1. ER Stress Is Induced by Palmitate and Contributes to the Increase of MCP-1 and IL-6 Expression in Mature Adipocytes

**Figure 1:**
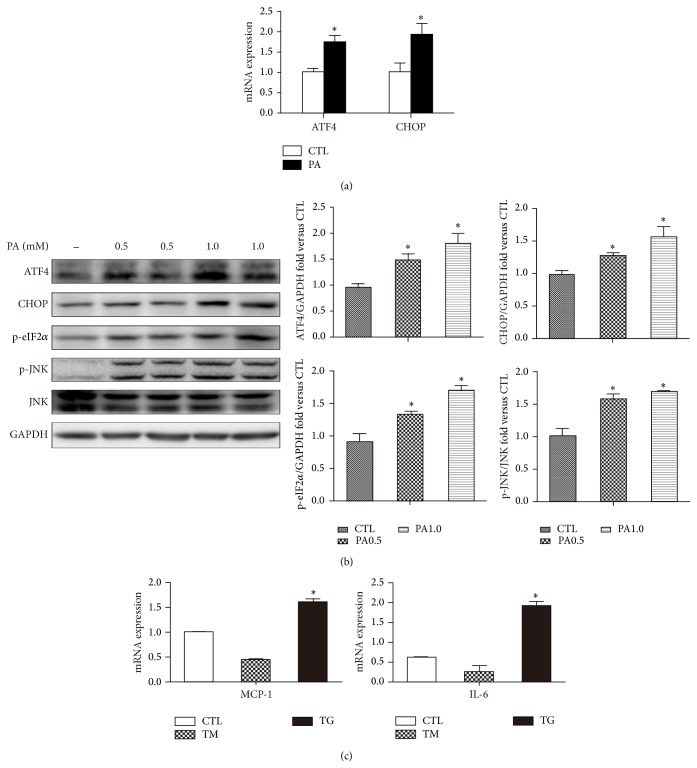
ER stress is induced by palmitate and contributes to increase MCP-1 and IL-6 expression. (a) Mature 3T3-L1 adipocytes were treated with 0.5% BSA or BSA-conjugated PA (0.5 mM) for 12 h. mRNA of ATF4 and CHOP measured by qPCR. (b) Western blot of indicated ER stress markers with or without PA (0.5 or 1.0 mM) treatment. Representative blots and quantifications are shown. (c) mRNA levels of MCP-1 and IL-6 with or without the ER stressors tunicamycin (TM, 1 *μ*g/mL) or thapsigargin (TG, 1 *μ*M) measured by qPCR. Results are mean ± SEM of three to five separate experiments. ^*^ *P* < 0.05 versus nontreatment group (0.5% BSA).

**Figure 2:**
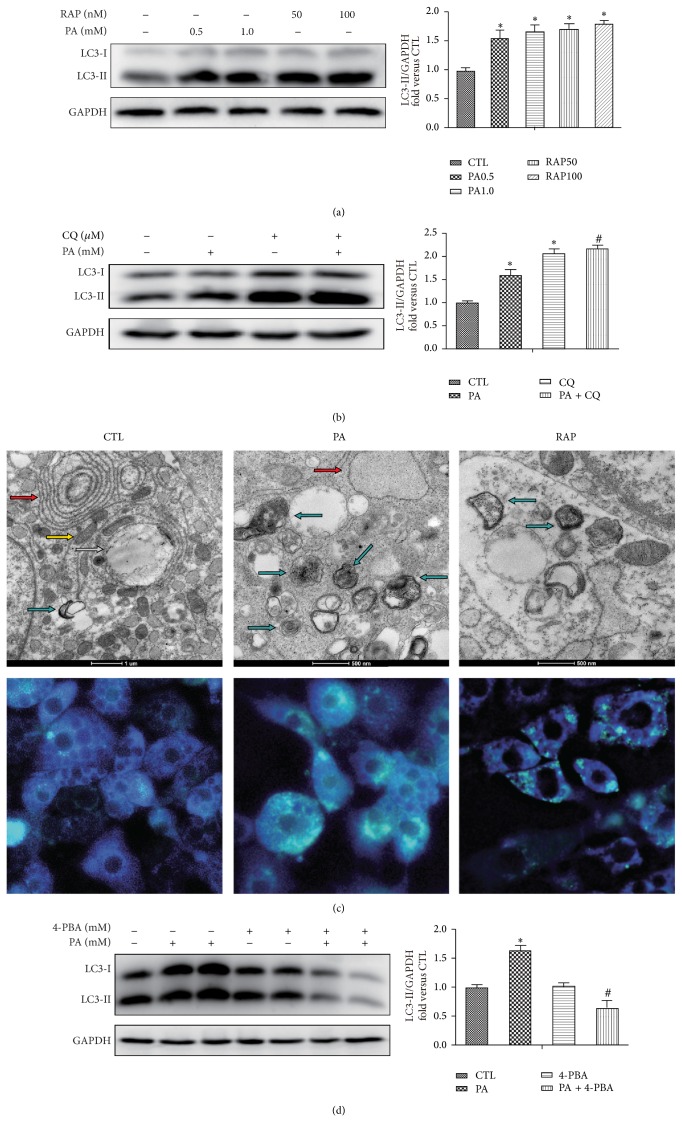
Autophagy is activated in response to palmitate and rapamycin. (a) Adipocytes were treated with or without palmitate (0.5 or 1.0 mM) and rapamycin (50 or 100 nM) for 12 h, followed by LC3 Western blotting analysis. (b) Preincubated with or without CQ (10 *μ*M) for 1 h and with BSA or BSA-conjugated PA for 12 h, followed by LC3 Western blotting analysis. (c) Electron microscopy and MDC staining of mature adipocytes expose to 0.5 mM PA or 100 nM RAP for 12 h. Lipid droplets (white arrow); autophagosomes and autolysosome of double-membrane, single-membrane, and multivesicular body-like vesicles (blue arrows); endoplasmic reticulum (red arrow); mitochondria (yellow arrow). Scale bars are indicated at bottom. (d) Pretreat with or without 4-PBA (10 mM) for 1 h and with PA (0.5 mM) for 12 h followed by LC3 Western blotting analysis. The proteins are quantified and normalized to GAPDH. Results are mean ± SEM of three to five separate experiments. ^*^ *P* < 0.05 versus nontreatment group (0.5% BSA); ^#^ *P* < 0.05 versus PA 0.5 mM group.

We investigated mRNA and protein expression of ER stress markers in PA-loaded adipocyte for 12 h treatment. The mRNA levels of ATF4 and CHOP were significantly increased compared with untreated cells (Figure 1(a)). Consistently, a corresponding increase in ER stress proteins markers was shown by an induction of ATF4, CHOP, and eIF2*α* as well as JNK phosphorylation with indicated PA dose (Figure 1(b)). These results suggested the activation of PERK-associated UPR pathways as well as IRE1 pathways. Increased CHOP expression occurs downstream of the main pathways activated following ER stress, namely PERK, ATF6, and IRE1 [17]. In addition, electron microscopy performed on PA-treated 12 h adipocytes showed numerous cells possessed dilated ER (Figure 2(c) PA group red arrow), which indicated ER stress compared with normal ER (Figure 2(c) CTL group red arrow).

ER stress has been linked to inflammatory responses and eventually cell death [18]. In this study, the ER stressor thapsigargin (TG) by depleted ER Ca^2+^ stores significantly increased the expression of MCP-1 and IL-6 in mature adipocytes, but this proinflammatory response was not observed with tunicamycin (TM), an agent that triggers the UPR by inhibiting protein glycosylation (Figure 1(c)).

### 3.2. Autophagy Is Activated in Response to Palmitate and Rapamycin in Mature Adipocytes

A reliable marker of autophagy is the conversion of the ATG protein LC3 from a soluble form (LC3-I) to a lipidized form (LC3-II), which stably associates with the membranes of autophagosomes [19]. This conversion can be detected by either observing the formation of punctuate structures or by measuring the accumulation of the LC3-II form. Western blot analysis showed an increase of LC3-II upon PA (0.5 and 1.0 mM) and rapamycin (RAP, 50 and 100 nM) for 12 h treatment (Figure 2(a)). Since accumulated LC3-II could be attributable to increased autophagosome formation or decreased lysosomal fusion and degradation, we next we use chloroquine (CQ), lysosome inhibitor, to the blockade of the autophagic flux [20]. The results showed that the pretreatment of CQ further promoted the accumulation of LC3-II in PA treat adipocytes (Figure 2(b)), which excluded the possibility of lysosomal dysfunction caused LC3-II accumulation.

To gain insight into the morphological changes of autophagy induced, electron microscopy was performed. Treatment with PA for 12 h induced the formation of autophagosomes, which was recognized at the ultrastructural level as double-membrane vacuolar structures containing visible cytoplasmic contents (Figure 2(c) PA group blue arrow). Autolysosomes which are recognized as single-membrane vacuolar structures containing high-density materials and some multivesicular body-like vesicles were appeared in cells expose to PA (Figure 2(c)). These features were also observed in cells with RAP (Figure 2(c), RAP group). In contrast, early autophagosomes were not abundant and there was rare evidence of autophagosomes or the autophagy pathway in nontreated cells (Figure 2(c) CTL group). Monodansylcadaverine (MDC) is known to accumulate specifically in autophagosomes or autophagic vacuoles (AV). A granular staining pattern indicates the formation of AVs [21], and the autophagosomes induced in this study were detected as green fluorescent granules in the cytoplasm, mainly around the nucleus, using fluorescence microscopy. Labeling of control adipocytes with MDC resulted predominantly in diffuse staining with only a few vacuoles taking up the stain (Figure 2(c)). In contrast, adipocytes exposed to PA and RAP showed extensive MDC accumulation in AVs 12 h treatment (Figure 2(c)).

We investigated the relationship between PA-induced ER stress and autophagy with ER stress inhibitor 4-phenyl butyrate (4-PBA). Western blot analysis of LC3-II accumulation was decreased in the presence of 4-PBA compared with PA alone (Figure 2(d)), indicating that increased autophagic flux induced by PA is at least in part response to ER stress.

### 3.3. Rapamycin Decreases PA-Induced ER Stress Pathways and Inflammatory Cytokines Expression

**Figure 3:**
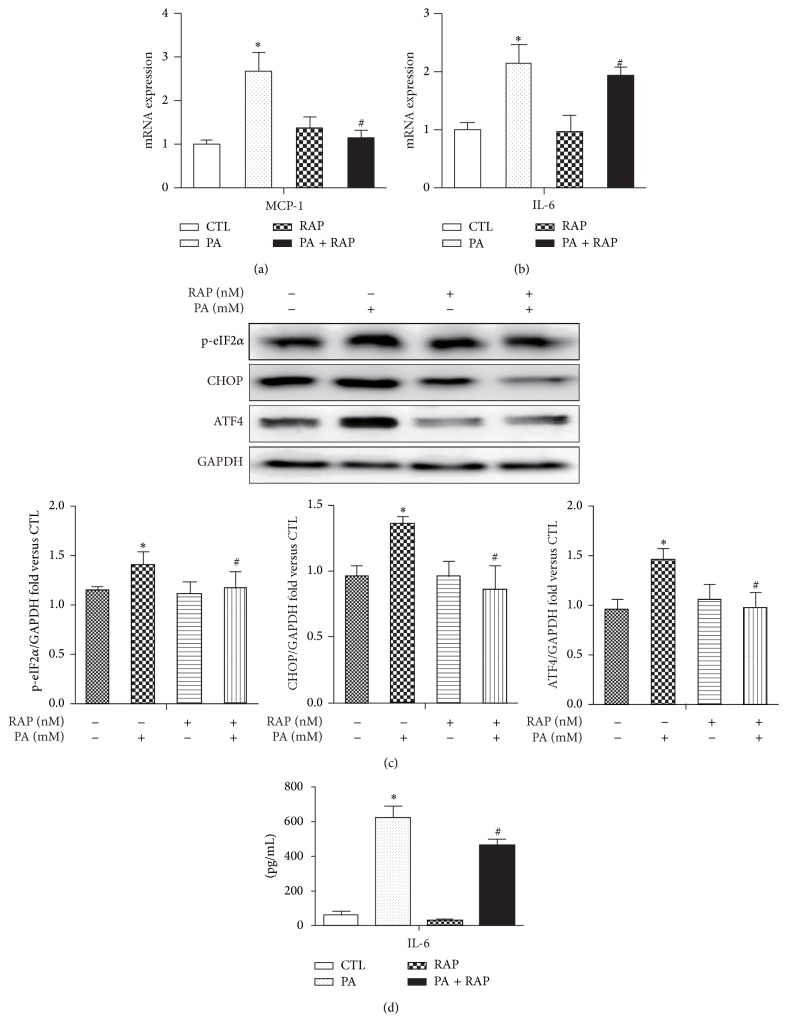
Rapamycin decreases PA-induced inflammatory cytokines and ER stress pathways. Mature 3T3-L1 adipocytes were pretreated with or without RAP (100 nM) for 1 h and with 0.5% BSA or BSA-conjugated PA (0.5 mM) for 12 h. (a, b) mRNA levels of MCP-1 and IL-6 were analyzed by qPCR. (c) Western blot and quantification for the protein expression with the indicated antibodies. (d) IL-6 secretion in mature 3T3-L1 adipocytes medium. Data are presented as mean ± SEM of three to five separate experiments. ^*^ *P* < 0.05 versus nontreatment group (0.5% BSA); ^#^ *P* < 0.05 versus PA 0.5 mM group.

**Figure 4:**
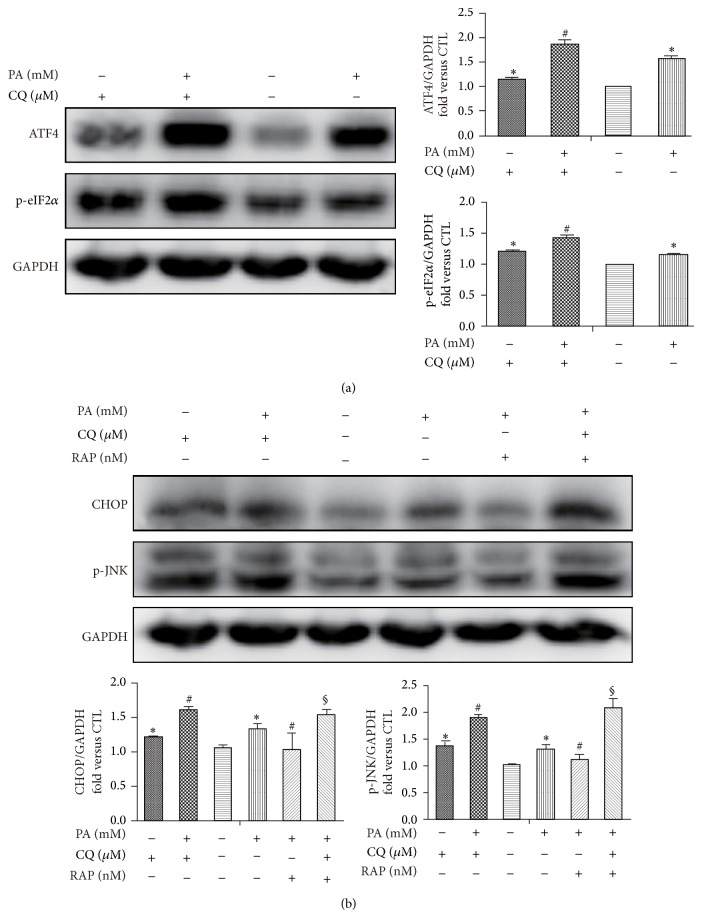
Rapamycin decreases PA-induced ER stress via stimulating autophagy. Mature 3T3-L1 adipocytes were pretreated with or without CQ (10 *μ*M) for 1 h and followed with PA (0.5 mM) and/or RAP (100 nM) for 12 h. Western blot and quantification for the protein expression with the indicated antibodies. Data are presented as mean ± SEM of three to five separate experiments. ^*^ *P* < 0.05 versus nontreatment group (0.5% BSA); ^#^ *P* < 0.05 versus PA 0.5 mM group; ^§^ *P* < 0.05 versus PA 0.5 mM with RAP 100 nM group.

The expression of inflammatory cytokines MCP-1 and IL-6 was induced by PA for 12 h compared with control by RT-PCR analysis. Pretreatment with rapamycin significantly reduced PA-induced MCP-1 and IL-6 mRNA expression as well as IL-6 secretion in medium (Figures 3(a), 3(b), and 3(d)), indicating rapamycin played a role in limiting PA-induced inflammation. In addition, rapamycin decreased PA-induced ER stress pathway, including PERK-associated UPR pathways as well as IRE1 pathways, evidenced by decreased protein levels eIF2*α* phosphorylation, ATF4, and CHOP (Figure 3(c)) as well as JNK phosphorylation (Figure 4(b)).

### 3.4. Rapamycin Decreases PA-Induced ER Stress via Stimulating Autophagy

To confirm that rapamycin effect on ERs was mediated via stimulating autophagy, we used CQ to the blockade of the autophagic flux. Pharmacological inhibition of autophagy by CQ significantly increased ER stress markers p-eIF2*α*, ATF4 (Figure 4(a)), and CHOP, as well as JNK phosphorylation (Figure 4(b)), indicating the central role of autophagy in regulating PA-induced ER stress. In addition, inhibition of autophagy itself by CQ was also shown with increased number of these ER stress markers, indicating basal autophagy may be important for controlling ER stress. Next, we examined whether stimulation of autophagy by rapamycin and reduction of ER stress were causally related. We cotreated mature adipocytes together with rapamycin and CQ and then studied the impact on ER stress. Inhibition of autophagy by CQ not only abolished the antistress effect of rapamycin and even exacerbated PA-induced ER stress, evidenced by further increased of CHOP and JNK phosphorylation (Figure 4(b)). Thus autophagy mediates the rapamycin protection of stressed adipocyte induced by palmitate.

### 3.5. Rapamycin Inhibits PA-Induced Inflammatory Gene Expression via Depressing NF*κ*B Pathways

**Figure 5:**
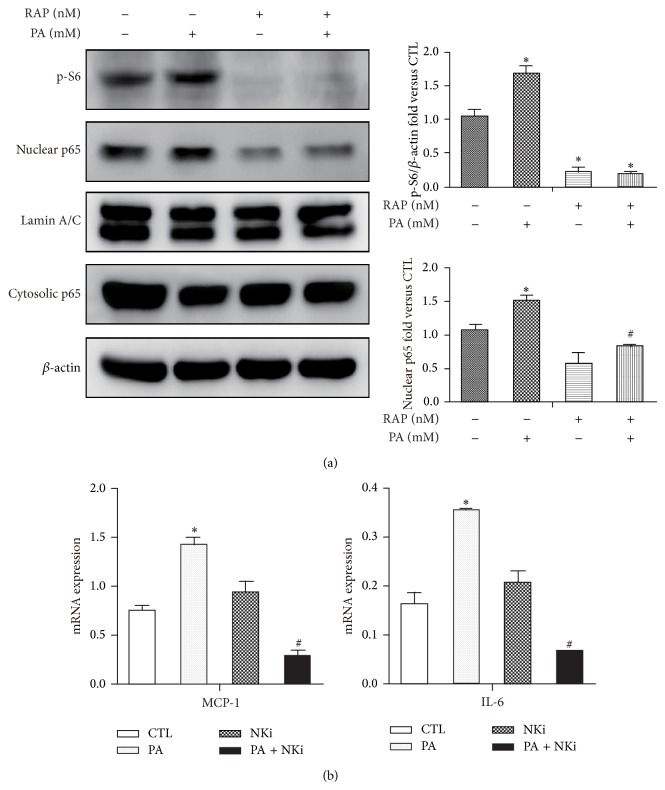
Rapamycin inhibits PA-induced inflammatory gene expression via depressing NF*κ*B pathways. (a) Mature 3T3-L1 adipocytes were pretreated with or without RAP (100 nM) and with 0.5% BSA or BSA-conjugated PA (0.5 mM) for 12 h. (a) Expression of nuclear and cytoplasmic p65 and p S6 are determined by Western blotting. Lamin/C and *β*-actin are included as loading controls. (b) Mature adipocytes were pretreatment with NKi (NF-*κ*B inhibitor, BAY11-7082 10 *μ*M) followed by PA treatment for 12 h. mRNA levels of cytokines and chemokines were determined by qPCR. Data are presented as mean ± SEM of three to five separate experiments. ^*^ *P* < 0.05 versus nontreatment group (0.5% BSA); ^#^ *P* < 0.05 versus PA 0.5 mM group.

Our finding that rapamycin treatment results in decreased inflammatory gene expression in PA-loaded adipocytes led us to hypothesis that rapamycin may play a role in controlling adipocytes inflammation in response to palmitate. Nuclear factor *κ*B dependent pathways are important for regulating inflammatory expressions in adipocytes. In the present study, PA treatment significantly induced the nuclear translocation of NF*κ*B P65 subunit in mature adipocytes by western blot analysis (Figure 5(a)). BaY11-7082 administration, a NF*κ*B inhibitor, abolished the mRNA expression of proinflammatory cytokines IL-6 and chemokines MCP-1 induced by PA (Figure 5(b)). These data indicated that PA activated IL-6 and MCP-1 is NF*κ*B-dependent. Pretreatment with rapamycin, followed by PA significantly attenuated the nuclear translocation of NF*κ*B P65 (Figure 5(a)), correlated with the decreased of MCP-1 and IL-6 mRNA expression as well as IL-6 secretion (Figure 3). In this study, PA-induced mTORC1 activity, evidenced by increase of phosphorylation S6 by western blot (Figure 5(a)), indicating that mTORC1 may be involved in the regulation of inflammation. Rap, as mTOR inhibitor, significantly inhibited phosphorylation S6 in both RAP group with or without PA (Figure 5(a)).

## 4. Discussion

Disruption of adipocytes homeostasis can be central in the inflammatory state, insulin resistance, and dyslipidemia [22]. The attenuation of ER stress decreases proinflammatory cytokines and alleviates downregulation of adiponectin [23], emphasizing that ER stress is involved in the dysfunction of adipocytes. The autophagy machinery is thought to have evolved as a stress response that allows cell survive during harsh conditions, probably by regulating energy homeostasis and/or by protein and organelle quality control [24]. Autophagy could involve in the modulation of inflammatory pathways, because autophagy has been implicated in immune cell function at multiple levels [25]. In our studies, we produce several findings: (1) autophagy is activated in response to PA depended on ER stress in mature adipocytes; (2) rapamycin ameliorates cellular ER stress via stimulating autophagy; (3) rapamycin dampens inflammatory cytokines expression via depressing NF*κ*B pathways.

Previous studies suggest abnormal autophagy and enhanced ER stress are interdependently involved in adipose tissue in type 2 diabetes [26]. Similarly, high-fat diet and obesity impaired autophagy in liver, resulting in ER stress and insulin resistance [11]. Impaired autophagic flux is associated with increased ER stress during the development of nonalcoholic fatty liver disease [27]. In our study, we investigated this interaction in mature adipocyte. Our results demonstrated that inhibiting autophagy further increased the sensitive of adipocyte to ER stress with or without PA treatment. Deficient autophagy serves to augment ER stress and promote further worsening of insulin resistance exposed to tunicamycin [26], a finding that is consistent with our results. These data confirm the antistress role of autophagy under various stress conditions. The impact of obesity on autophagy remains controversial. Autophagy was found suppressed both* in vitro* and in the adipose tissue of high-fat-fed mice in Yoshizaki et al.'s study [28]. In contrast, other studies demonstrated that autophagy was upregulated in adipose tissue during obesity with a positive correlation with BMI (body mass index) [29]. The autophagosome content was also shown to be increased in isolated adipocytes derived from obese and diabetic humans [30]. We observed increased autophagy flux in response to palmitate, which was at least in part ER stress dependent. Our previous study demonstrated that palmitate is shown to elicit ER stress-JNK-autophagy axis. Previous study suggests that ER stress may be a source of the membranes during the formation of autophagic vesicles. ER stress mediates the polyglutamine-induced LC3 conversion, as essential step for autophagy formation [31]. ER stress negatively regulates AKT/TSC/mTOR pathway to enhance autophagy [32]. In addition, it has been postulated that ER stress-induced autophagy may evolve as a mechanism to dispose of misfolded proteins that cannot be degraded by endoplasmic reticulum related degradation (ERAD), consequently assisting ER homeostasis [32, 33]. Thus ER stress-induced autophagy may be as a positive response to cell apoptosis and cellular stress at early stage, while persistence ER stress may disrupt autophagy and contributes to cell dysfunction and cell death [27].

The mammalian target of rapamycin (mTOR) serves as an important regulator of autophagy. Under nutrient availability, mTORC1 phosphorylates Atg13, which prevents binding to Atg1 (ULK1 in mammals) and hence reduced formation of the Atg1-Atg13-Atg17 complex [34]. Conversely, mTORC1 inhibition by rapamycin administration stimulates initiation of autophagosome budding. In this study, we obverse that rapamycin augments autophagy and protected against the development of ER stress in the context of PA-loaded adipocytes. In Akita *β* cells, the inhibition of ER stress by rapamycin is not mediated via reducing proinsulin load, but though stimulating autophagy [13]. Stimulation of autophagy by rapamycin improves ER stress and apoptosis in Akita *β* cells and islets, evidenced by decreased CHOP expression and JNK phosphorylation [13]. Restoring autophagic flux by rapamycin prevented PA-induced PERK, eIF2*α*, and JNK phosphorylation in human hepatic cells [27]. Thus, we hypothesize rapamycin attenuating ER stress is via stimulating autophagy in this context. Indeed, inhibiting autophagy abolished the antistress effect of rapamycin and even augments PA-induced adipocyte ER stress to certain extent. These findings strongly suggest the positive effect of rapamycin on cell stress is depended on autophagy.

Decrease of IL-6 and MCP-1 expression by rapamycin indicates the role of rapamycin on dampening PA-induced inflammatory cytokines in mature adipocytes. NF*κ*B and JNK dependent pathways regulated cytokines release from artificially hypertrophied 3T3-L1 adipocytes [35]. In addition, PA-induced mTORC1 activity may be involved in inflammation. Activation of mRORC1 augments protein synthesis, which may increase the ER client protein load, leading to exacerbation of ER stress [36]. ER stress interacts with many different inflammatory signaling pathways [7]. In this study, MCP-1 and IL-6 expression was increased by TG but not TM, which may underlie the importance of ER Ca^2+^ depletion in the cross talk between UPR and inflammation. Many investigations focus on the role of autophagy on inflammation, and rapamycin, like other analogues, is not only a potent inducer of autophagy but has anti-inflammation actions as well. Such as sirolimus downregulates LPS-induced expression of chemokines in monocytes, such as MCP-1 by inhibiting the NF*κ*B-p65 and MAPK-P38 signaling pathways [37]. Importantly, we found that with the inhibition of mTORC1 by rapamycin was correlated with the decreased of inflammatory cytokines and signalling pathway NF*κ*B. Rapamycin in macrophages protects mice against inflammation and insulin resistance potentially by inhibiting HFD- and palmitic acid-induced IRE1*α*/JNK/NF*κ*B pathways activation [38]. However, distinguishing an independent effect of rapamycin on inflammation rather than on autophagy may be difficult. Recently it has been suggested that autophagy and inflammation are intimately linked. Previous study demonstrated autophagy somehow act to limit excessive inflammatory gene expression. Inhibition of autophagy in human and mouse adipose tissue explants led to a significant increase in IL-1*β*, IL-8 mRNA expression and protein secretion [29]. In macrophages a TOR-autophagy spatial coupling compartment (TASCC) augments cellular function and facilitates the mass synthesis of secretory proteins, such as cytokines [39]. Disruption of the TASCC suppressed the synthesis of IL-6 and IL-8 in macrophages [39]. Thus the concomitant effects of rapamycin on autophagy and inflammation may occur though autophagy-dependent cytokine synthesis.

In conclusion, we believe our data support the idea that rapamycin decreases PA-induced ER stress/NF-*κ*B signaling pathway, thereby inhibiting inflammatory cytokines associated with autophagy activation. Perhaps the combination of cell stress adaptation and immune response modulation with induction of autophagy is what will ultimately prove beneficial targeting a single mechanistic pathway. Although rapamycin is unsuitable for diabetes treatment due to its multiple side effects, previous research suggests that, in some context, stimulation of autophagy by rapamycin overrides the deleterious effects of the drug [13]. Stimulating autophagy by rapamycin may become a way to attenuate adipocytes dysfunction.

## Figures and Tables

**Table 1:** Primers used for real-time PCR.

Gene	Forward primer (5′-3′)	Reverse primer (5′-3′)
IL-6	CTGGGAAATCGTGGAAATG	CCAGAGGAAATTTTCAATAGGC
MCP-1	AGCCAACTCTCACTGAAGCCA	AGTAGCAGCAGGTGAGTGGG
ATF4	GGACAGATTGGATGTTGGAGAAAATG	GGAGATGGCCAATTGGGTTCAC
CHOP	GTCCAGCTGGGAGCTGGAAG	CTGACTGGAATCTGGAGAG
36B4	CGACCTGGAAGTCCAACTAC	ATCTGCTGCATCTGCTTG
